# Adiponectin and insulin resistance are related to restenosis and overall new PCI in subjects with normal glucose tolerance: the prospective AIRE Study

**DOI:** 10.1186/s12933-019-0826-0

**Published:** 2019-03-04

**Authors:** Ferdinando Carlo Sasso, Pia Clara Pafundi, Raffaele Marfella, Paolo Calabrò, Federico Piscione, Fulvio Furbatto, Giovanni Esposito, Raffaele Galiero, Felice Gragnano, Luca Rinaldi, Teresa Salvatore, Michele D’Amico, Luigi Elio Adinolfi, Celestino Sardu

**Affiliations:** 1 Department of Advanced Medical and Surgical Sciences, University of Campania “Luigi Vanvitelli”, Piazza Miraglia 2, 80138 Naples, Italy; 2 University of Campania “Luigi Vanvitelli”, Piazza Miraglia 2, 80138 Naples, Italy; 3 Division of Cardiology, A.O.R.N. Sant’Anna e San Sebastiano, Caserta, Italy; 4 Department of Translational Medical Sciences, University of Campania “Luigi Vanvitelli”, Naples, Italy; 5 Department of Medicine and Surgery, University of Salerno, Via Allende, 84081 Baronissi, SA Italy; 6 Department of Cardiology, Cardarelli Hospital, Via Antonio Cardarelli 9, 80131 Naples, Italy; 7 Department of Advanced Biomedical Sciences, University of Naples “Federico II”, Via Pansini 5, 80131 Naples, Italy; 8 Department of Experimental Medicine, University of Campania “Luigi Vanvitelli”, Via Santa Maria di Costantinopoli 16, 80138 Naples, Italy

**Keywords:** Percutaneous coronary intervention, Insulin resistance, Adipokines, Restenosis, Glucose tolerance

## Abstract

**Background:**

In patients with Normal Glucose Tolerance (NGT) some causes of ischemic heart disease (IHD) were not completely investigated. The role both of metabolic *milieu* and adipokines in IHD progression was not fully investigated. Our aim was to assess the link between adipokines plasma levels, insulin resistance (IR) and IHD in NGT patients undergoing Percutaneous Coronary Intervention (PCI).

**Methods:**

AIRE is a single-center prospective longitudinal observational study investigating the IHD outcome of NGT subjects who underwent coronary revascularization by PCI in a third level cardiology center at A.O. dei Colli Hospital, University of Campania “Luigi Vanvitelli”. Six hundred seventy-nine subjects hospitalized in 2015 for coronary arteriography not suffering from Acute Coronary Syndrome (ACS) in the previous 4 weeks, as well as from all conditions could affect glycemic plasma levels and IR status, were assessed for eligibility. Fifty-four patients with neither history of diabetes nor Altered Fasting Glucose (AFG)/Impaired Fasting Glucose (IGT) after Oral Glucose Tolerance Test (OGTT) were finally enrolled. Primary endpoint was the assessment of the relationship of adipokines and HOMA-IR with the occurrence of restenosis in NGT subjects. As secondary endpoint we assessed the association of the same adipokines and IR with overall ACS events after PCI in NGT subjects.

**Results:**

The 54 NGT patients enrolled were mainly males (85%), with a median age of 60 years [IQR 58–63 years]. Only 4 patients (7.4%) experimented restenosis. Median follow-up was equal to 29.5 months [IQR 14.7–34 months]. Adiponectin levels were independently associated to restenosis (OR 0.206; 95% CI 0.053–0.796; p = 0.000). Instead HOMA-IR and adiponectin appeared independently associated both to de novo IHD (OR 9.6*10^13^; 95% CI 3.026–3.08*10^27^; p = 0.042 and OR 0.206; 95% CI 0.053–0.796; p = 0.000, respectively) and overall new PCI (OR 1.5*10^11^; 95% CI 2.593–8.68*10^21^; p = 0.042 and OR 0.206; 95% CI 0.053–0.796; p = 0.000, respectively). Moreover, we fixed a potential cut-off for adiponectin for risk of restenosis (≤ 8.5 µg/mL) and overall new PCI (≤ 9.5 µg/mL).

**Conclusion:**

IR and cytokines play a role in progression of any stage of IHD also in NGT subjects. Our results in this setting of patients, though the relatively small sample size, represent a novelty. Future studies on larger populations are needed to analyze more in depth adipokines and insulin resistance role on IHD progression in non-diabetic people.

**Electronic supplementary material:**

The online version of this article (10.1186/s12933-019-0826-0) contains supplementary material, which is available to authorized users.

## Background

Cardiovascular disease (CVD) represents the most common cause of death among adult people [1]. Therefore, it is important to reassess cardiovascular risk factors and evaluate new strategies to improve CVD prognosis. When considering metabolic risk factors for CVD, emphasis is usually placed on the role played by insulin resistance (IR) [2]. In fact, IR shows a cluster of abnormalities which might increase the risk of ischemic heart disease (IHD) [2].

To date, several studies confirmed the central role played by IR in enhancing the CVD risk in prediabetes and insulin-resistant individuals, with a relative risk approximately twice higher than non-insulin resistant subjects [3–6]. On the other hand, a reduced insulin resistance by altered inflammation/oxidative stress may even be found in patients with Normal Glucose Tolerance (NGT), and they still bear increased risks for cardiovascular disease [7, 8]. Insulin resistance is usually measured through the HOMA IR index, which takes into account both fasting glycemia and insulinemia [9]. Recent analysis of a wide cohort showed substantial similarities in the inflammatory profiles as well as insulin-resistance background associated with DM and CVD, confirming a “common soil” which needs to be investigated also in patients with normal glucose tolerance [10]. Therefore, it is intriguing to evaluate the relationship between inflammation/oxidative stress and cytokines milieu and IHD in NGT patients. In this setting, recent advances established a fundamental role for inflammation as an important component of IHD [11], with a clear association existing between inflammatory cytokines relapsed by adipose tissue, inflammation and atherosclerosis [12–14]. Indeed, adipose tissue secretes many biologically active mediators such as TNF-α, plasminogen activator inhibitor type 1 (PAI-1), IL-6, leptin, adiponectin and resistin [15]. Among these, known as adipokines, particularly adiponectin seems to be directly connected to IHD [16]. Intriguingly, adiponectin is the unique adipokine exclusively secreted from adipose tissue which improves insulin sensitivity and favorably modulates the endothelial inflammatory response to vascular injury [17]. Through its insulin-sensitizing, anti-inflammatory and anti-atherogenic properties, lower plasma levels of adiponectin can be therefore IHD independent predictors [18, 19]. Thereafter, most recent studies on NGT patients showed that neointimal tissue proliferation after Percutaneous Coronary Intervention (PCI) was greater in Impaired Glucose Tolerant (IGT) patients than in NGT patients at follow-up, and that the best predictor of restenosis was insulin level after oral glucose tolerance test (OGTT) [20, 21]. Finally, as relationship between type 2 diabetes mellitus (T2DM) and IR/adipokines has been widely investigated, our attention was focused on NGT patients. Therefore, aim of this paper was to assess the link between adipokines plasma levels, insulin resistance and IHD in NGT patients undergoing PCI, and particularly the relationship between adipokines/IR and restenosis, as well as overall Acute Coronary Syndrome (ACS) after PCI.

## Methods

### Design of the study

A single center prospective longitudinal observational study investigated the IHD outcome of NGT subjects who underwent coronary revascularization by PCI. Inclusion criteria were: age > 18 years, elective indication to receive a PCI in patients with suspect of IHD. Instead, subjects were considered not eligible if they suffered from Acute Coronary Syndrome (ACS) in the previous 4 weeks, in the case of recent steroid administration (last 3 months), and chronic inflammatory diseases (all these conditions could affect glycemic plasma levels as well as IR). Moreover, alcohol or drug assumption, known psychiatric/neoplastic diseases were further exclusion criteria, as well as known DM, altered fasting glycaemia (AFG) or an Impaired Glucose Tolerance (IGT) response at standard OGTT.

The study consisted in two phases. During the screening/enrollment phase, all patients hospitalized in a third level cardiology center at the A.O. dei Colli Hospital, University of Campania “Luigi Vanvitelli” in Naples for an elective coronary angiography for suspected IHD in 2015 were evaluated for eligibility. Eligible patients underwent OGTT, with blood samples taken at time 0′ and 120′ after oral 75 grams glucose administration for plasma glucose and insulin assay, as well as glycated hemoglobin (HbA1c), total HDL/cholesterol, triglycerides, creatinine, adiponectin, resistin and TNF-alpha dosage. To avoid potential influences on cytokines and IR, coronary angiography in election was performed at least 24 h after blood sampling. Only subjects with neither history of diabetes nor AFG/IGT after OGTT were enrolled in the study. Moreover, from enrollment and during entire follow-up all patients were under optimized medical therapy: ACE inhibitors, beta-blockers, statin and antiplatelets. The follow-up phase, instead, consisted in periodically scheduled visits after 3, 6 and 12 months from PCI. During each visit, anamnestic data, blood pressure and standard ECG were recorded. In addition, at 12 months patients also underwent cardiological examination (stress test) and blood sampling. Subsequently, the follow-up was annually performed clinically or by phone. A new coronary angiography at follow-up was performed in the case of new symptoms onset or evidence of ischemia/coronary disease progression at non-invasive imaging tests (including stress test or coronary computed tomography angiography). The use of additional invasive tests to assess coronary lesions characteristic (quantitative coronary analysis [QCA], intravascular ultrasound [IVUS], optical coherence tomography [OCT], fractional flow reserve [FFR]) was strongly recommended, but left to the discretion of the operator. All subjects gave written informed consent to participate to the study. The protocol was approved by the Ethical Committee of University of Campania.

### Endpoints of the study

Primary endpoint was the assessment of the relationship of adipokines, particularly adiponectin, resistin and TNF-alpha, and IR with the occurrence of clinically-driven target lesion revascularization due to in-stent restenosis (TLR-IS) in NGT subjects. TLR-IS was defined as either repeat percutaneous or surgical revascularization for a lesion anywhere within the stent, including focal, extended, totally occluding lesions.

As secondary endpoint, instead, we assessed the association of the same adipokines and IR with overall ACS events after PCI in NGT subjects (re-PCI and new PCI).

### PCI procedure

PCI was performed according to the current AHA/ACC guidelines [22], through radial or femoral approach using small size arterial sheaths. The analyses of all angiographic data were performed by interventional cardiologists and followed by PCI with angioplasty and drug eluting stent (DES) in case of critical angiographic stenosis [23]. Informed consent was obtained from each patient before coronary angiography. All patients received heparin (50 IU/kg intravenously) before the PCI. Angiographic lesion morphology was classified according to the AHA/ACC classification [22]. All PCIs were performed with second generation DES implantation. At clinical follow-up the restenosis was treated either with balloon angioplasty or new DES implantation, whilst PCI of new stenosis was performed exclusively with DES. All patients were on aspirin (100 mg daily) and clopidogrel (75 mg daily for 12 months) treatment after coronary artery stenting.

### Biochemical assays

#### ELISA tests

Plasma samples were collected using either heparin or EDTA as anticoagulant. They were centrifuged for 15 min at 1000×*g* within 30 min of collection and stored at − 20 °C until assayed. Plasma TNF-alpha and adiponectin levels were determined using commercially available human ELISA tests, from R&D systems (D6050, DTA00 and DRP300 respectively, Abingdon, UK) and following the manufacturer’s instructions; whilst plasmatic resistin levels with a commercially human ELISA assay from Ray Biotechinc (ELH-Resistin-1 Norcross, GA). These kits show negligible cross-reactivity (< 0.5%) and high sensitivity (detection limit 0.891 ng/mL for adiponectin; 2 pg/mL for resistin and 5.5 pg/mL for TNF-alpha, data by the manufacturer).

### Statistical analysis

Categorical variables were presented as number and percentages, whilst continuous variables were instead presented as median and interquartile range [IQR]. The normal/not normal distribution was preliminarily assessed through a Kolmogorov–Smirnov Goodness-of-Fit K-S test. Categorical variables were compared using the Chi square test, as the sample size was > 50 subjects, while the Mann–Whitney U Test and the Wilcoxon test were used for continuous variables. All variables emerged as significant at the univariate analysis were furtherly included in a logistic regression model to assess if there was a parameter independently associated with the occurrence of coronary events, adjusting for HbA1c, hypertension, smoke and age. The risk of re-PCI/new PCI was analyzed using the Kaplan–Meier (K–M) method, and the log-rank test was used to compare the cumulative risk between the two groups. A two-sided probability P value < 0.05 was considered statistically significant. In addition, ROC Curves were performed in order to find a potential cut-off for the three cytokines analyzed (TNF-alpha, resistin and adiponectin). The found cut-offs were used to dichotomize the cytokines and perform a further K–M analysis in order to assess the risk of PCI according to the level of each cytokine. All analyses were performed using SPSS statistical software (Version 24.0, SPSS Chicago, Il, USA)and R software (CRAN ^®^ 3.3.4).

## Results

### General characteristics and follow-up of the study population

**Fig. 1:**
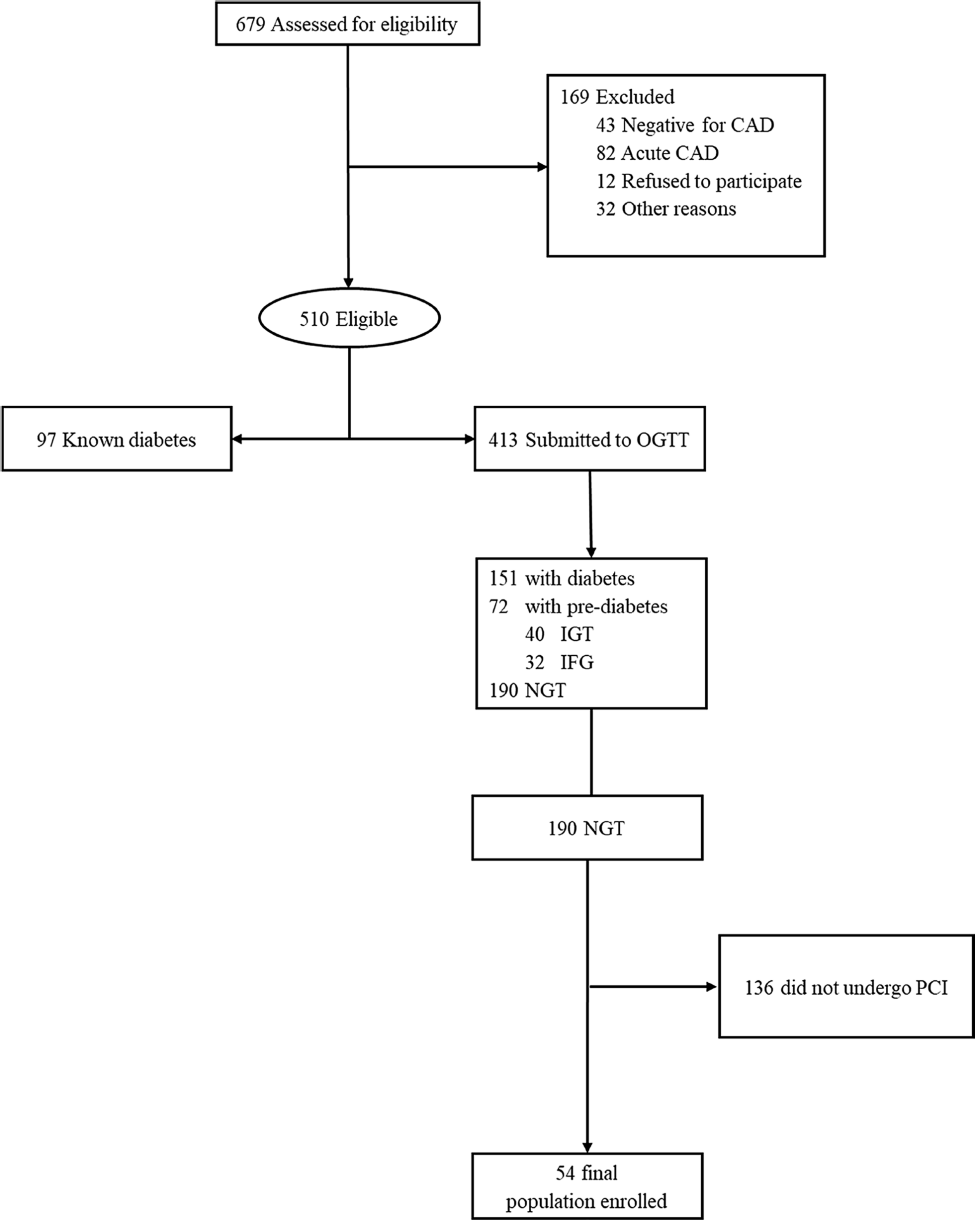
Flow-chart of the study

Six hundred seventy-nine subjects hospitalized for coronary arteriography were assessed for eligibility, 169 did not meet inclusion criteria. Among the 510 eligible, 97 were excluded due to diabetes mellitus (DM) history and 223/413 after OGTT (151 DM and 72 pre-DM). Therefore, only 190 were found NGT. However, due to the mandatory inclusion criteria, 136 NGT were further excluded, for a final enrolled population of 54 patients (Fig. 1). Anthropometric, clinical and biochemical characteristics are shown in Table 1. In particular, they were mainly males (46 cases; 85%), with a median age of 60 years [IQR 58–63 years] and a median BMI of 27 kg/m^2^ [IQR 25.5–28.1 kg/m^2^]. All patients presented a median fasting glycemia at baseline equal to 87 mg/dL [IQR 80–90.2 mg/dL], a median glycated hemoglobin (HbA1c) of 5.2% [IQR 5.1–5.5%] and the 80% were smokers. Median follow-up was equal to 29.5 months [IQR 14.7–34 months]. In 4 patients we observed the occurrence of re-stenosis (median time-from-event: 8.5 months [IQR 4–15.75 months]). Three patients with restenosis of previous PCI of LAD and one case of restenosis of right coronary artery were observed. Two of these restenosis of LAD involving DES implanted in long lesion of distal part of coronary with diameter 2.5 mm. The other restenosis of LAD involving a previous DES implantation in bifurcation with diagonal branch, while the only restenosis of right coronary artery involving previous PCI in distal part of coronary with small diameter. Of note, no stent thrombosis occurred during follow-up. In five cases, instead, de novo coronary lesions were reported at coronary angiography during follow-up, and in one case treated with PCI. All new stenosis were focal lesions significantly worse than those found in the previous coronary angiography (median time from event: 29 months [IQR 12–33.5 months]). All new PCI were performed either after positive echo-stress or in case of an ACS. Moreover, three subjects suffering from angina with no troponin elevation nor echo-stress with signs of inducible ischemia did not undergo PCI and were strictly clinically followed. None subject experienced different major cardiovascular events (CV death, fatal or no fatal stroke, amputation). Overall death was equal to the 2% (1 case), due to lung cancer.

**Table 1:** General characteristics at baseline and clinical findings during follow-up of the study population (n = 54)

Parameter	
Age (years), median [IQR]	60 [58–63]
Sex, no. (%)	
M/F	46 (85)/8 (15)
BMI, kg/m^2^, median [IQR]	27 [25.5–28.1]
Blood pressure (mmHg), median [IQR]	
Systolic	130 [120–131.2]
Diastolic	80 [70–80]
Hypertension, no. (%)	15 (28)
Smoke, no. (%)	43 (80)
Cholesterol (mg/dL), median [IQR]	
Total	172 [149.5–189]
HDL	42.5 [35–51]
LDL	103 [68–128]
Triglycerides (mg/dL), median [IQR]	127 [105.2–151.5]
Glycemia (mg/dL), median [IQR]	
Baseline	87 [80–90.2]
2 h	109 [93–126.7]
Glycated hemoglobin (%), median [IQR]	5.2 [5.1–5.5]
Insulin (µU/mL), median [IQR]	3.8 [3–6]
HOMA IR, median [IQR]	0.78 [0.59–1.31]
Creatinine (mg/dL), median [IQR]	1 [0.87–1.1]
Hemoglobin (mg/dL), median [IQR]	14.3 [13.3–15.2]
Adiponectin (µg/mL), median [IQR]	11 [9.7–13.2]
Adiponectin, no. (%)	
Pathologic	13 (24)
Resistin (ng/mL), median [IQR]	7 [4–9]
Resistin, no. (%)	
Pathologic	8 (15)
TNF-alpha (ng/mL), median [IQR]	8.5 [7–11]
TNF-alpha, no. (%)	
Pathologic	6 (11)
Site of stenosis at first PCI, no. (%)	
IVA	25 (46)
CX	10 (19)
DX	19 (35)
Follow-up duration (months), median [IQR]	29.5 [14.7–34]
Re-PCI, no. (%)	4 (7)
Time re-PCI, median [IQR]	8.5 [4–15.75]
Site of re-PCI, no. (%)	
IVA	3 (75)
CX	0 (−)
DX	1 (25)
New PCI, no. (%)	5 (9)
Time new PCI, median [IQR]	29 [IQR 12–33.5]
Site of new-PCI, no. (%)	
IVA	2 (40)
CX	1 (20)
DX	2 (40)
Death, no. (%)	
Cardiovascular/other causes	− /1 (2)

### Restenosis risk assessment: a univariate and multivariate analysis

**Fig. 2:**
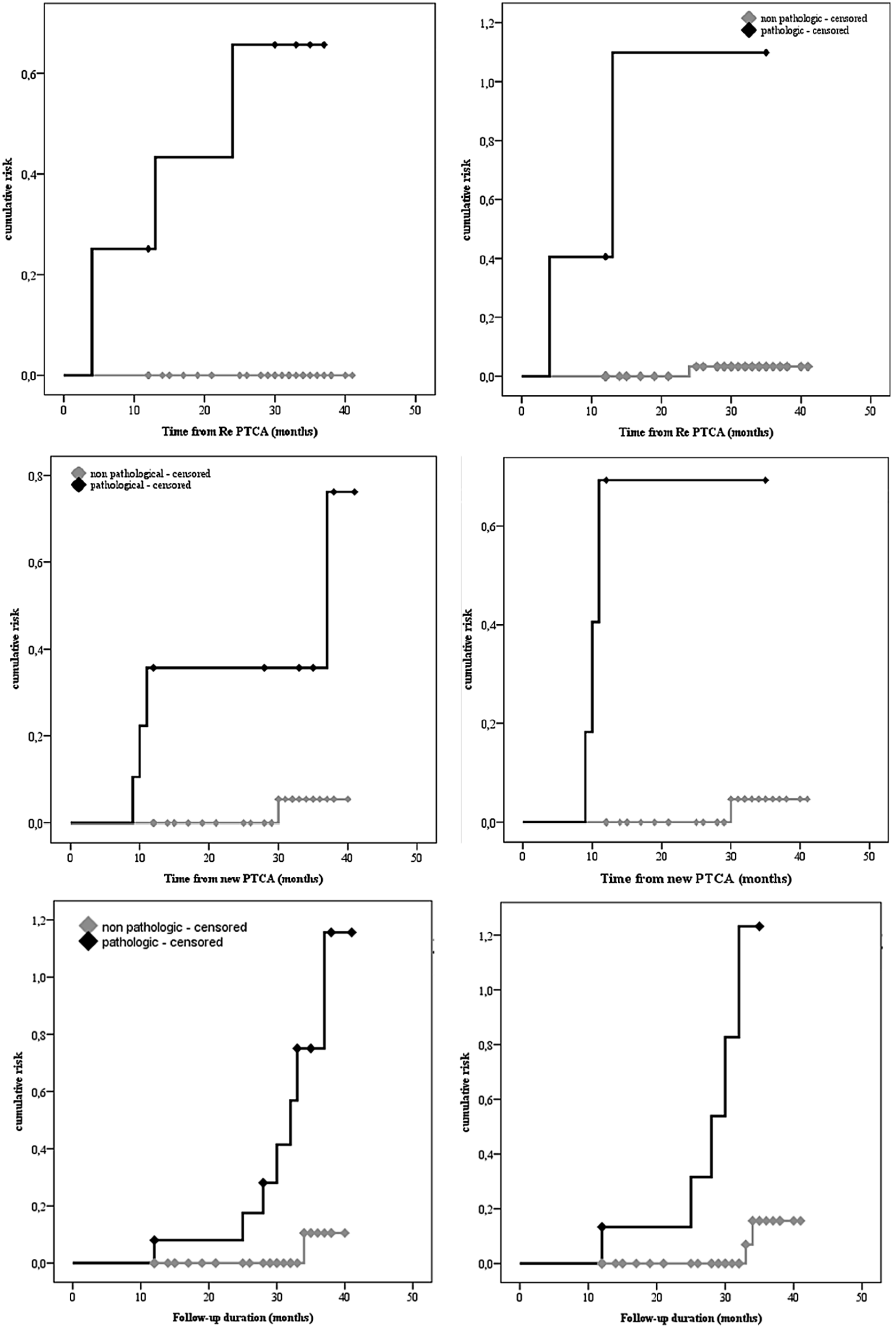
(Upper panel) K–M curves describing the risk of re-stenosis according to the values of the analyzed cytokines. Respectively adiponectin (left) and TNF-alpha (right). (Central panel) K–M curves describing the risk of new stenosis according to the values of the analyzed cytokines. Respectively adiponectin (left), resistin (right). (Lower panel) K–M curves describing the risk of any coronary event according to the values of the analyzed cytokines. Respectively adiponectin (left), resistin (right)

We assessed the distribution of the collected variables according to the occurrence or not of restenosis. At univariate analysis, restenosis events resulted significantly associated with higher levels of insulin (median 9.8 µU/mL vs 3.6 µU/mL; p = 0.003) and HOMA-IR (median 2.23 vs 0.75; p = 0.003), as well as higher glucose levels at 120 min (median 132.5 mg/dL vs 106 mg/dL; p = 0.021). Higher TNF-alpha levels revealed significantly associated with the development of restenosis (median 18.5 ng/mL vs 8 ng/mL; p = 0.008); the same in the case of lower adiponectin values (median 7 vs 11.5 µg/mL; p = 0.000). As expected, also time-from-event was significantly lower in subjects who experimented re-PCI (median 8.5 vs 29.5 months; p = 0.019). Only adiponectin levels demonstrated independently associated at the multivariate analysis (OR 0.206; 95% CI 0.053–0.796; p = 0.000). We also performed ROC Curves (Additional file 1: Fig. S1—upper panel) to find a potential cut-off for the three cytokines analyzed. For adiponectin, the AUROC was highly accurate (AUROC 0.965; p = 0.002; 95% CI 0.913–1.000) and allowed us to fix a potential cut-off at8.5 µg/mL (sensitivity 100%; specificity 90%), hence, values ≤ 8.5 µg/mL could be considered pathological. Resistin AUROC was instead equal to 0.730, not reaching the statistical significance. Instead, TNF-alpha AUROC was 0.878 (p = 0.013; 95% CI 0.709–1.000) and the found cut-off was around 14 ng/mL (sensitivity 75%; specificity ≈ 94%). Values > 14 ng/mL could hence be defined as pathological. Thus, we dichotomized adiponectin and TNF-alpha and performed a K–M analysis to evaluate the cumulative risk of restenosis according to the level of cytokine. For both the cytokines analyzed the log-rank p was highly statistically significant (p = 0.000) (Fig. 2—upper panel).

### Risk assessment of de novo IHD: a univariate and multivariate analysis

We also assessed the occurrence or not of new stenosis, excluding patients who had restenosis (n = 51). At the univariate analysis de novo IHD revealed significantly associated with higher levels of insulin (median 9.8 µU/mL vs 3.5 µU/mL; p = 0.008) and HOMA-IR (median 2.34 vs 0.74; p = 0.012), as well as higher glucose levels both at baseline and at 120 min (median 97 mg/dL vs 86.5 mg/dL; p = 0.038 and median 135 mg/dL vs 102.5 mg/dL; p = 0.045, respectively) and HbA1c levels (median 5.6% vs 5.2%; p = 0.001). Differently from restenosis risk, higher levels of TNF-alpha did not reveal significantly associated with the development of new; conversely in the case of higher resistin levels (median 15 ng/mL vs 6 ng/mL; p = 0.012) and in the case of lower values of adiponectin (median 8 vs 12 µg/mL; p = 0.001). Only HOMA-IR (OR 9.6*10^13^; 95% CI 3.026–3.08*10^27^; p = 0.042) and adiponectin appeared independently associated at the multivariate analysis (OR 0.206; 95% CI 0.053–0.796; p = 0.000). All data are shown in Additional file 2: Table S1. Also in this case we performed ROC Curves (Additional file 1: Fig. S1—central panel). Adiponectin AUROC expressed a high accuracy (AUROC 0.911; p = 0.003; 95% CI 0.814–1.000), hence we fixed a potential cut-off of 9.5 (sensitivity 80%; specificity 87%); hence, values ≤ 9.5 could be considered pathological. TNF-alpha AUROC was equal to 0.607 and did not reach the statistical significance; instead, for resistin AUROC was 0.833 (p = 0.015; 95% CI 0.555–1.000) and the found cut-off around 10.5 (sensitivity 80%; specificity ≈ 96%). Values > 10.5 could hence be defined as pathological. Dichotomized adiponectin and resistin were analyzed by a K–M analysis to evaluate the cumulative risk of new stenosis. For both the cytokines the log-rank p reached a high statistical significance (log-rank p = 0.002 and log-rank p = 0.000, respectively) (Fig. 2—central panel) (Table 2).

**Table 2:** Characteristics of the study population, according to the occurrence or not of restenosis (n = 54)

Parameter	Univariate analysis			Multivariate analysis	
	Re-PCI		p	OR [95% CI]	p
	Yes (n = 4)	No (n = 50)			
Age (years), median [IQR]	59.5 [58.2–60.7]	60 [57.7–63]	0.714		
Sex, no. (%)			0.386		
M	4 (100)	42 (84)			
F	0 (−)	8 (16)			
BMI, kg/m^2^, median [IQR]	25.3 [24.8–26.7]	27.2 [25.8–28.1]	0.159		
Blood pressure (mmHg), median [IQR]					
Systolic	130 [115–137.5]	130 [120–131.2]	1.000		
Diastolic	80 [72.5–80]	80 [70–80]	0.714		
Hypertension, no. (%)	1 (25)	14 (28)	0.897		
Smoke, no. (%)	4 (100)	39 (78)	0.293		
Cholesterol (mg/dL), median [IQR]					
Total	157 [147.5–180.7]	174.5 [147.5–192]	0.555		
HDL	34.5 [31.7–41]	43 [35–51.2]	0.066		
LDL	90.5 [86–116]	109 [70.2–122.5]	0.874		
Triglycerides (mg/dL), median [IQR]	141.5 [121.5–166.7]	125.5 [103–151.5]	0.327		
Glycemia (mg/dL), median [IQR]					
Baseline	93.5 [86.2–102.2]	87 [79.7–90]	0.066		
2 h	132.5 [117.7–136.7]	106 [93–123]	0.021		
Glycated hemoglobin (%), median [IQR]	5.6 [5.3–5.8]	5.2 [5–5.5]	0.051		
Insulin (µU/mL), median [IQR]	9.8 [9.4–10]	3.6 [2.8–4.7]	0.003		
HOMA IR, median [IQR]	2.23 [2.1–2.4]	0.75 [0.57–0.97]	0.003		
Creatinine (mg/dL), median [IQR]	1 [1–1.16]	1 [0.8–1.1]	0.266		
Hemoglobin (mg/dL), median [IQR]	14.2 [13.2–14.5]	14.3 [13.3–15.2]	0.534		
Adiponectin (µg/mL), median [IQR]	7 [6–8]	11.5 [10–14]	0.000	0.206 [0.053–0.796]	0.022
Resistin (ng/mL), median [IQR]	13 [5.2–15.5]	6.5 [4–8.2]	0.137		
TNF-alpha (ng/mL), median [IQR]	18.5 [11–20.7]	8 [7–10]	0.008		
Time RE-PCI, median [IQR]	8.5 [4–21.2]	29.5 [13.5–34]	0.019		
Follow-up duration (months), median [IQR]	30 [25.7–32.7]	29.5 [13.5–34]	1.000		
Death, no. (%)					
Cardiovascular/other causes	0 (−)/−	0 (−)/1 (2)	0.775		

### Risk assessment of overall new PCI: a univariate and multivariate analysis

Finally, we assessed the overall occurrence or not of coronary events. At the univariate analysis coronary events resulted significantly associated with higher levels of insulin (median 9.8 vs 3.5 µU/mL; p = 0.000) and HOMA-IR (median 2.23 vs 0.74; p = 0.000), as well as higher glucose levels both at baseline and at 120 min (median 95.5 vs 86.5 mg/dL; p = 0.015 and 132 vs 102.5 mg/dL; p = 0.007) and HbA1c levels (median 5.6% vs 5.2%; p = 0.001). Higher levels of resistin revealed significantly associated with the development of coronary events (median 13 vs 6 ng/mL; p = 0.013); the same in the case of lower adiponectin values (median 8 vs 12 µg/mL; p = 0.000). At the multivariate analysis HOMA IR (OR 1.5*10^11^; 95% CI 2.593–8.68*10^21^; p = 0.042) and adiponectin levels appeared independently associated (OR 0.206; 95% CI 0.053–0.796; p = 0.000). All data are shown in Table 3. Even in this case, we performed ROC Curves (Additional file 1: Fig. S1—lower panel). For adiponectin, the AUROC expressed a high accuracy (AUROC 0.931; p = 0.000; 95% CI 0.857–1.000) and we fixed a potential cut-off at 9.5 µg/mL (sensitivity 87.5%; specificity 87%), with values ≤ 9.5 µg/mL considered as pathological. For what concerns TNF-alpha the AUROC was 0.700 and did not reach the statistical significance; instead resistin AUROC was 0.772 (p = 0.015; 95% CI 0.513–1.000) and we fixed the cut-off at 10.5 ng/mL (sensitivity 75%; specificity ≈ 96%). Values > 10.5 ng/mL could hence be defined as pathological. Thus, we dichotomized both cytokines and performed a K–M analysis, with respect to the entire follow-up period, to evaluate the cumulative risk of coronary events. For both the log-rank p reached a high statistical significance (log-rank p = 0.001 and log-rank p = 0.000, respectively) (Fig. 2—lower panel).

**Table 3:** Characteristics of the study population, according to the occurrence or not of overall new PCI (for restenosis and de novo IHD) (n = 54)

Parameter	Univariate analysis			Multivariate analysis	
	Overall new PCI		p	OR [95% CI]	p
	Yes (n = 8)	No (n = 46)			
Age (years), median [IQR]	59.5 [58–60.7]	60.5 [57.7–63]	0.590		
Sex, no. (%)			0.380		
M	6 (75)	40 (87)			
F	2 (25)	6 (13)			
BMI, kg/m^2^, median [IQR]	26.7 [24.8–28.8]	27 [25.8–28]	1.000		
Blood pressure (mmHg), median [IQR]					
Systolic	125 [120–130]	130 [120–136.2]	0.296		
Diastolic	80 [72.5–80]	80 [70–80]	0.574		
Hypertension, no. (%)	1 (14)	14 (30)	0.296		
Smoke, no. (%)	7 (88)	36 (78)	0.549		
Cholesterol (mg/dL), median [IQR]					
Total	173.5 [147.5–209.7]	172.5 [146.2–189]	0.658		
HDL	44 [34.2–64.5]	42 [35–51]	0.590		
LDL	107.5 [86–123.7]	105 [70.2–119.7]	0.765		
Triglycerides (mg/dL), median [IQR]	127.5 [117.2–157.7]	127 [102.7–151.5]	0.495		
Glycemia (mg/dL), median [IQR]					
Baseline	95.5 [86.2–97.7]	86.5 [79.7–89.2]	0.015	0.643 [0.389–1.064]	0.086
2 h	132 [116–136.7]	102.5 [93–121.2]			
Glycated hemoglobin (%), median [IQR]	5.6 [5.5–5.7]	5.2 [5–5.4]	0.001		
Insulin (µU/mL), median [IQR]	9.8 [9.1–10.1]	3.5 [2.8–4.3]	0.000	0.008 [0.000–1.183]	0.058
HOMA IR, median [IQR]	2.23 [2.1–2.5]	0.74 [0.57–0.94]	0.000	1.5*10^11^ [2.593–8.68 * 10^21^]	0.042
Creatinine (mg/dL), median [IQR]	1 [0.85–1.1]	1 [0.87–1.1]	0.971		
Hemoglobin (mg/dL), median [IQR]	14.2 [13.2–14.8]	14.3 [13.3–15.3]	0.495		
Adiponectin (µg/mL), median [IQR]	8 [6.5–8.7]	12 [10–14]	0.000	0.206 [0.053–0.796]	0.022
Resistin (ng/mL), median [IQR]	13 [6–15]	6 [4–8]	0.013		
TNF-alpha (ng/mL), median [IQR]	13 [7.5–20.7]	8 [7–10]	0.073		
Follow-up duration (months), median [IQR]	31 [25.7–33.7]	29 [13.5–34]	0.558		
Death, no. (%)					
Cardiovascular/other causes	0 (−)/−	0 (−)/1 (2)	0.674		

## Discussion

It is well known that coronary artery disease progresses more rapidly in diabetic subjects. In particular insulin treated diabetes is an independent and strong predictor of late and repeat coronary revascularization [24]. Intriguingly, in the general population treated with PCI, insulin resistance promoted the “late catch-up phenomenon”, a restenosis due to continuous neointimal growth during long-term follow-up, after first generation DES implantation [25]. Indeed, the Casale Monferrato study suggested that, insulin resistance was associated to natriuretic peptides serum levels, with lower circulating levels in insulin-resistance non diabetic people, and higher values in the upper spectrum of metabolic abnormalities, such as diabetes with the metabolic syndrome [26]. These findings seem indirectly to be confirmed by the observation that in patients undergoing elective PCI, pioglitazone, an insulin-sensitizer drugs, improved cardiometabolic profiles. In particular, pioglitazone increased adiponectin and reduced plasma levels of natriuretic peptides [27]. Therefore, growing evidences indicate that adiposity and impaired glycemia levels importantly contribute to IHD even in non-diabetic individuals [28]. To date, visceral as well as epicardial adiposity are related to ischemic heart diseases through secretion of adipokines and pro-inflammatory factors from adipocytes [29]. Actually, the role of adipokines on IHD in NGT subjects is still not well-known. In a previous cross-sectional study, both number of stenosed coronary arteries and coronary damage score were found independently associated to fasting and post load insulin and HOMA in NGT subjects [7]. Moreover, the present study refers to a longitudinal analysis on non-obese NGT patients submitted to PCI. In this setting, we observed that metabolic and inflammatory factors are significantly related to relapse of new PCI, due to both restenosis and de novo stenosis. Restenosis constitutes an important limitation after stent implantation, but its pathogenesis is not yet fully understood. Several studies showed increased HOMA-IR levels as an important prognostic indicator in non-diabetic patients who underwent PCI [30–32]. Free survival from IHD in non-diabetic patients treated with primary PCI is significantly correlated with plasma glucose levels at admission [33], and elevated glucose levels on admission are associated with an adverse prognosis in patients with STEMI treated with PCI [34]. On the other hand, the metabolic milieu and, above all, IR and cytokines levels are strongly influenced by the adrenergic stress and inflammatory status occurring during an ACS. Therefore, glucose tolerance, IR and cytokines levels during ACS are not representative of their status before and a few days after an ACS. These last conditions could similarly impact the CV outcome in subjects with IHD (Additional file 3: Table S2).

In non-diabetic patients, neointimal tissue proliferation after stent implantation is greater in IGT patients than in NGT patients at 6 months of follow-up. Moreover, higher insulin and HOMA levels are suggested as the best predictors of restenosis [35]. More recently, insulin resistance was associated with in-stent restenosis in no diabetic patients undergoing coronary DES implantation at long-term angiographic follow-up [36]. Some reports investigated the significance of serum adiponectin levels in predicting clinical outcomes after PCI. These studies demonstrated that, in male patients with acute myocardial infarction (AMI), adiponectin blood concentration was independently predictive of AMI during a 1-year follow-up after primary PCI [21]. Originally, our study suggests that pre-procedural lower expression of adiponectin may be an independent predictor of major cardiovascular events after PCI. In particular, patients with significantly lower pre-procedural adiponectin serum levels had higher rate of AMI than those with higher levels. Univariate analysis suggests that risk of restenosis in patients with NGT was significantly associated with higher levels of insulin and HOMA-IR. Moreover, higher TNF-alpha levels revealed significantly associated with the development of restenosis, and the same occurred in the case of lower values of adiponectin. However, only adiponectin levels appear independently associated with the occurrence of restenosis at the multivariate analysis. This last finding is most likely due to the small sample size of our population. Overall new PCI (restenosis and de novo IHD) emerged significantly associated with higher levels of insulin and HOMA-IR, whilst higher levels of resistin were significantly associated with the development of coronary events, and the same in the case of lower adiponectin values. At the multivariate analysis, HOMA IR and adiponectin levels revealed independently associated. Our findings are consistent with An et al. who found IR as an independent predictor of atherosclerosis plaque progression in patients with IHD, both in DM and non-DM patients [37]. Since adiponectin has insulin-sensitizing, anti-inflammatory and anti-atherogenic properties, low plasma levels of adiponectin can be therefore independent predictors of IHD, and platelet activation in carotid atherosclerosis both in diabetic and non-diabetic subjects [38–41]. In addition, excessive inflammatory coronary responses may be related to a lack of this adipocytokine [19, 42, 43]. Moreover, adiponectin revealed as a potential biological marker for restenosis in T2DM patients and, in this sense, it has been tried to find a possible cut-off (plasma adiponectin levels ≥ 6.0 µg/mL) associated with a lower risk of restenosis both in diabetic and non-diabetic subjects [44]. In the general population, none cut-off has been found yet. However, it has been recently demonstrated an association between lowest adiponectin serum levels and an increased risk of restenosis due to endothelium functions impairment [45]. Actually, no data is available in NGT people about resistin role on atherosclerosis development. To date, only few studies show that resistin levels are correlated with cardiovascular death in patients with documented IHD, i.e. unstable angina and myocardial infarction [19]. Intriguingly, it was demonstrated that resistin levels are associated with increased restenosis rates in diabetic patients with IHD after successful coronary stenting [46]. Indeed, pre-procedural serum resistin concentrations were higher in the restenosis group rather than in the patients without restenosis [46]. This association was found in the general population too. Originally, our data, included in a Kaplan–Meier analysis, showed a statistically significant association between pathological values of the cytokines and a higher risk of overall new PCI in NGT. Finally, results from our study are also useful to remark how the use optimal medical therapy, including potent antiplatelet agents, should be implemented in patients undergoing PCI as they have proven to be of benefit regardless of concomitant risk factors or glycemic/diabetic status [47].

## Study limitations

This study presents several limitations. First of all, we have to mention the small sample size of our population, which affects the results of the multivariate analysis. To date, this might be particularly evident for what concerns HOMA-IR index, which loses of significance at the multivariate analysis. Actually, the sample size might be justified by the difficult to find higher numbers of patients NGT subjects who underwent coronary revascularization by PCI for IHD. Another study limitation is the short duration of 2.5 years of follow-up. This might be a limiting factor to assess at longer follow up duration the important clinical effects of the present study analysis. On the other hand, the overall assessment of new PCI seems to strengthen our results and confirm previous cross-sectional studies in NGT subjects.

## Conclusions

In conclusion, our findings strongly support the role of IR and cytokine in progression of any stage of IHD also in people without a metabolic disease (diabetes or prediabetes) as NGT subjects. Future studies are needed to analyze more in depth and on larger populations the role of adipokines and IR on IHD progression in non-diabetic people. In particular, a pharmacological treatment of this metabolic and inflammatory milieu should be studied in order to try to improve the CV outcomes of these large setting of patients.

## Additional files

**Additional file 1: Figure S1.** Definition of the best cut-off level of ROC curves respectively for adiponectin (a), resistin (b) and TNF-alpha (c), according to the risk of restenosis. (Central panel) Definition of the best cut-off level of ROC curves respectively for adiponectin (a), resistin (b) and TNF-alpha (c), according to the risk of new stenosis. (Lower panel) Definition of the best cut-off level of ROC curves respectively for adiponectin (a), resistin (b) and TNF-alpha (c), according to the risk of any coronary event.

**Additional file 2: Table S1.** Characteristics of the study population, according to the occurrence or not of PCI for de novo IHD (n = 51).

**Additional file 3: Table S2.** Adiponectin and HOMA-IR values for the patients who experimented restenosis.

## Abbreviations

CVD: cardiovascular disease
IR: insulin resistance
IHD: ischemic heart disease
NGT: normal glucose tolerance
PAI-1: plasminogen activator inhibitor type 1
PCI: percutaneous coronary intervention
OGTT: oral glucose tolerance test
T2DM: type 2 diabetes mellitus
DM: diabetes mellitus
ACS: Acute Coronary Syndrome
AFG: altered fasting glycaemia
IGF: impaired glucose tolerance
DES: drug eluting stent
IQR: interquartile range
K–M: Kaplan–Meier
HbA1c: glycated hemoglobin
AMI: acute myocardial infarction
