# Supplementary figures and images for: Adiponectin and insulin resistance are related to restenosis and overall new PCI in subjects with normal glucose tolerance: the prospective AIRE Study
Source: Cardiovasc Diabetol. 2019 Mar 4;18:24. doi: 10.1186/s12933-019-0826-0 (PMC6399947; doi:10.1186/s12933-019-0826-0)

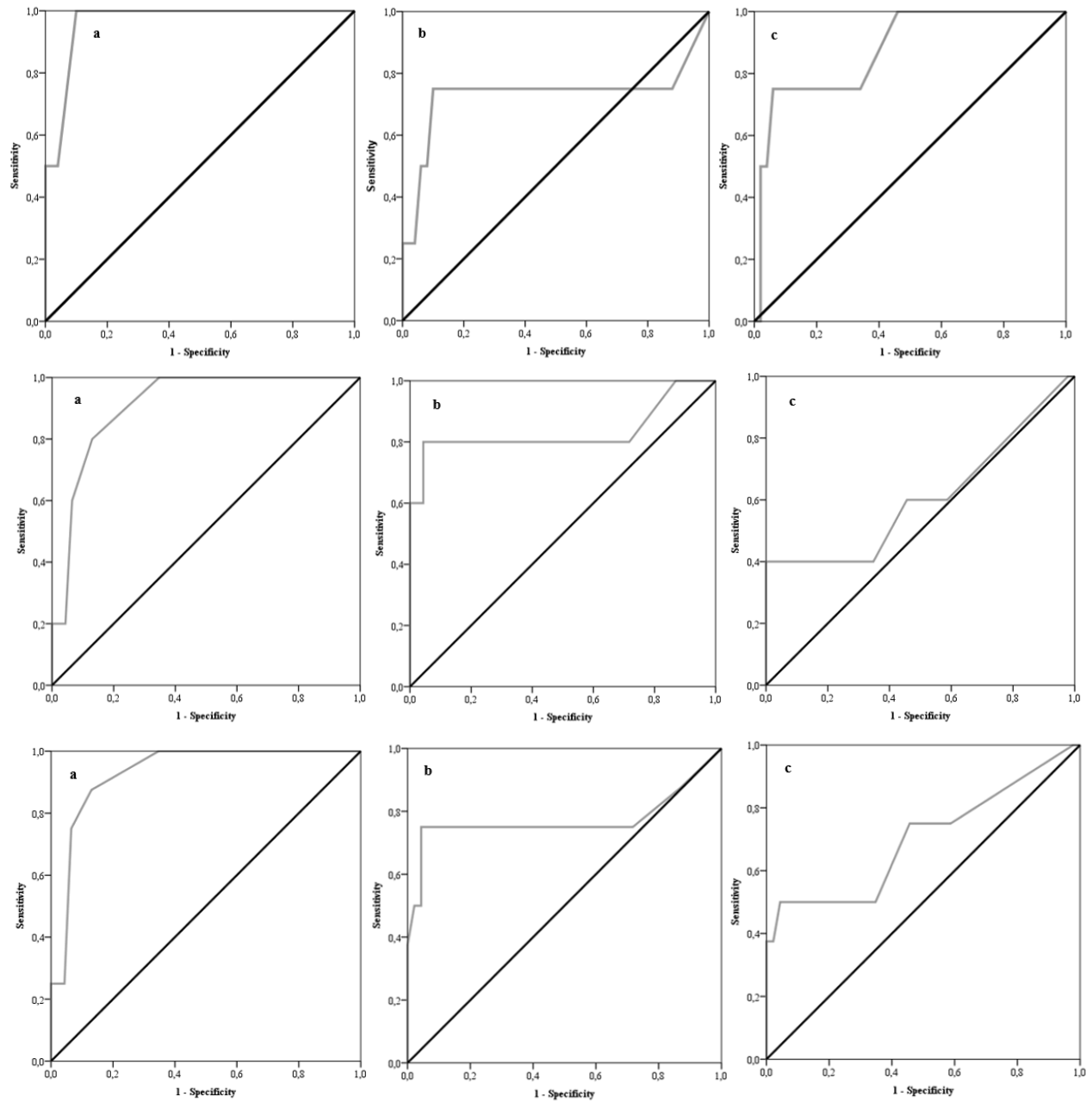

Supplement: Supplementary file 1 — Additional file 1: Figure S1. Definition of the best cut-off level of ROC curves respectively for adiponectin (a), resistin (b) and TNF-alpha (c), according to the risk of restenosis. (Central panel) Definition of the best cut-off level of ROC curves respectively for adiponectin (a), resistin (b) and TNF-alpha (c), according to the risk of new stenosis. (Lower panel) Definition of the best cut-off level of ROC curves respectively for adiponectin (a), resistin (b) and TNF-alpha (c), according to the risk of any coronary event. [file 12933_2019_826_MOESM1_ESM.tif]
