# Supplementary material for: Adiponectin and insulin resistance are related to restenosis and overall new PCI in subjects with normal glucose tolerance: the prospective AIRE Study
Source: Cardiovasc Diabetol. 2019 Mar 4;18:24. doi: 10.1186/s12933-019-0826-0 (PMC6399947; doi:10.1186/s12933-019-0826-0)
Supplement: Supplementary file 2 — Additional file 2: Table S1. Characteristics of the study population, according to the occurrence or not of PCI for de novo IHD (n = 51). [file 12933_2019_826_MOESM2_ESM.docx]

| **Supplementary Table 1.** Characteristics of the study population, according to the occurrence or not of PCI for de novo IHD (n=51). | | | | | |
| --- | --- | --- | --- | --- | --- |
| **Parameter** | **Univariate Analysis** | | | **Multivariate Analysis*** | |
|  | **PCI for de novo IHD** | |  |  |  |
|  | **Yes (n=5)** | **No (n=46)** | **p** | **O.R. [95% C.I.]** | **p** |
| Age (years), median [IQR] | 60 [56.5 – 62.5] | 60.5 [57.7 – 63] | 0.747 |  |  |
| Sex, No. (%)  *M*  *F* | 3 (60)  2 (40) | 40 (87)  6 (13) | 0.115 |  |  |
| BMI, kg/m2, median [IQR] | 28.4 [24.5 – 28.9] | 27 [25.8 – 28] | 0.945 |  |  |
| Blood Pressure (mmHg), median [IQR]  *Systolic*  *Diastolic* | 130 [120 – 130]  80 [70 – 80] | 130 [120 – 136.2]  80 [70 – 80] | 0.939  0.218 |  |  |
| Hypertension, No. (%) | 0 (-) | 14 (30) | 0.148 |  |  |
| Smoke, No. (%) | 4 (80) | 36 (78) | 0.928 |  |  |
| Cholesterol (mg/dL), median [IQR]  *Total*  *HDL*  *LDL* | 188 [146 – 220.5]  54 [39.5 – 70]  120 [64 –136] | 172.5 [146.2 –189]  42 [35 – 51]  105 [70.2 – 119.7] | 0.449  0.091  0.612 |  |  |
| Triglycerides (mg/dL), median [IQR] | 123 [112.5 – 146] | 127 [102.7 – 151.5] | 0.890 |  |  |
| Glycemia (mg/dL), median [IQR]  *Baseline*  *2h* | 97 [84 – 101]  135 [106.5 – 137.5] | 86.5 [79.7 – 89.2]  102.5 [93 – 121.2] | 0.038  0.045 | 0.616 [0.355 – 1.069] | 0.085 |
| Glycated hemoglobin (%), median [IQR] | 5.6 [5.5 – 5.8] | 5.2 [5 – 5.4] | 0.001 |  |  |
| Insulin (µU/mL), median [IQR] | 9.8 [6.2 – 11.8] | 3.5 [2.8 – 4.3] | 0.008 | 0.002 [0.000 – 1.113] | 0.054 |
| HOMA IR, median [IQR] | 2.34 [1.4 – 2.9] | 0.74 [0.57 – 0.94] | 0.012 | 9.6*1013 [3.026 – 3.08*1027] | 0.042 |
| Creatinine (mg/dL), median [IQR] | 1 [0.7 – 1.15] | 1 [0.87 – 1.1] | 0.842 |  |  |
| Hemoglobin (mg/dL), median [IQR] | 14 [11.8 – 14.9] | 14.3 [13.3 – 15.3] | 0.449 |  |  |
| Adiponectin (µg/mL), median [IQR] | 8 [7 – 9.5] | 12 [10 – 14] | 0.001 | 0.206 [0.053–0.796] | 0.022 |
| Resistin (ng/mL), median [IQR] | 15 [8 – 15.5] | 6 [4 – 8] | 0.012 |  |  |
| TNF-alpha (ng/mL), median [IQR] | 9 [6.5 – 21.5] | 8 [7 – 10] | 0.449 |  |  |
| Time new-PCI, median [IQR] | 11 [9.5 – 33.5] | 29 [13.5 – 34] | 0.127 |  |  |
| Follow-up duration (months), median [IQR] | 30 [20 – 35.5] | 29 [13.5 – 34] | 0.701 |  |  |
| Death, No. (%)  *Cardiovascular / Other causes* | 0 (-)/ - | 0 (-)/1 (2) | 0.739 |  |  |
| * multivariate analysis was adjusted for HbA1c, hypertension, smoke and age. | | | | | |
