# Supplementary material for: Adiponectin and insulin resistance are related to restenosis and overall new PCI in subjects with normal glucose tolerance: the prospective AIRE Study
Source: Cardiovasc Diabetol. 2019 Mar 4;18:24. doi: 10.1186/s12933-019-0826-0 (PMC6399947; doi:10.1186/s12933-019-0826-0)
Supplement: Supplementary file 3 — Additional file 3: Table S2. Adiponectin and HOMA-IR values for the patients who experimented restenosis. [file 12933_2019_826_MOESM3_ESM.docx]

| **Supplementary Table 2.** Adiponectin and HOMA-IR values for the patients who experimented restenosis. | | |
| --- | --- | --- |
| **Patient** | **HOMA** | **Adiponectin** |
| **C.A.** | **2,52** | **6** |
| **R.G.** | **2,23** | **8** |
| **T.F.** | **2,24** | **6** |
| **V.A.** | **2,06** | **8** |
